# 2-Oxo-2*H*-chromen-7-yl penta­noate

**DOI:** 10.1107/S241431462500937X

**Published:** 2025-10-28

**Authors:** A. J. N’Gouan, H. Bazié, S. D. Goulizan Bi, A. Djandé, R. Kakou-Yao, C. Lecomte

**Affiliations:** a Laboratory of Material, Sciences, Environnement and Solar Energy, Research Team: Crystallography and Molecular Physics, University Félix Houphouêt-Boigny, 22 BP 582 Abidjan 22, Côte d’Ivoire; b Laboratory of Molecular Chemistry and Materials, Research Team: Organic Chemistry and Phytochemistry, University Joseph KI-ZERBO, 03 BP 7021, Ouagadougou 03, Burkina Faso; c Laboratory of Molecular Chemistry and Materials, Research Team:, Organic Chemistry and Phytochemistry, University Joseph KI-ZERBO, 03 BP 7021, Ouagadougou 03, Burkina Faso; d CRM2, CNRS-Université de Lorraine, Vandoeuvre-lès-Nancy CEDEX BP 70239, France; University of Antofagasta, Chile

**Keywords:** crystal structure, hydrogen bond, coumarins, Hirshfeld surface

## Abstract

In the title compound, C_14_H_14_O_4_, the dihedral angle between the coumarin nucleus and the penta­noate moiety is 62.20 (7)°. The coumarin moiety is planar as usual, with a maximum deviation from the least-squares plane of 0.081 (2) Å. In the crystal, mol­ecules are linked by C—H⋯O hydrogen bonds into centrosymmetric dimers with an *R*_2_^2^(8) graph-set motif, and the cohesion of the crystal is also supported by π–π inter­actions with a centroid–centroid distance of 3.9342 (8) Å. A Hirshfeld surface analysis revealed that 44.6% of the inter­molecular inter­actions are from H⋯H contacts, 28.2% are from ⋯O/O⋯H contacts and 16.3% are from H⋯C/C⋯H.

## Abstract 2

In the title compound, C_14_H_14_O_4_, the dihedral angle between the coumarin nucleus and the penta­noate moiety is 84.6 (9)°. In the crystal, mol­ecules are linked by C—H⋯O hydrogen bonds into centrosymmetric dimers with an *R*_2_^2^(8) graph-set motif, and π–π inter­actions are also observed.

## Graphical abstract

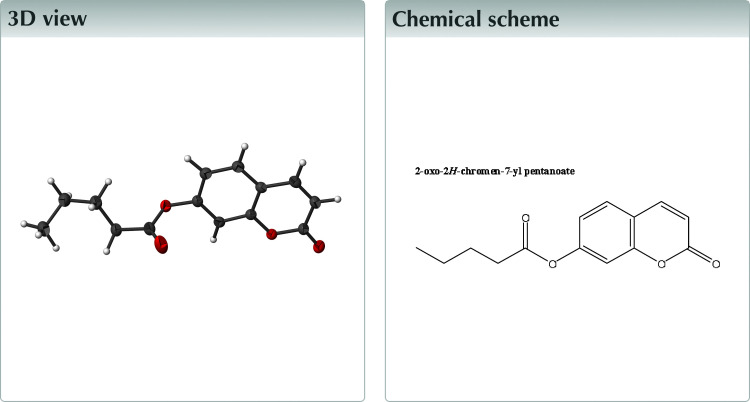

## Structure description

**Figure 1:**
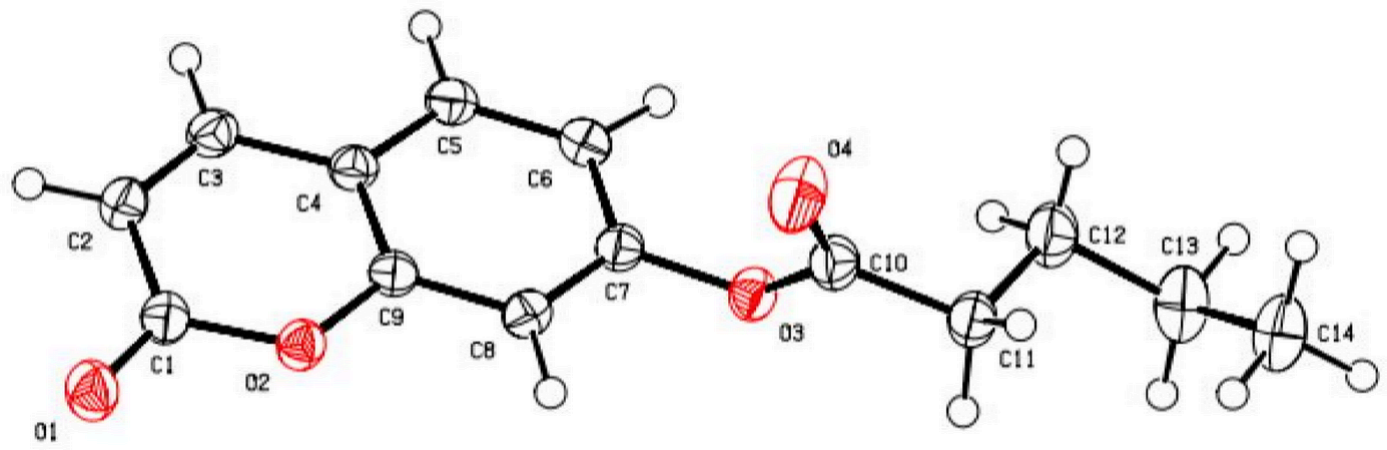
The title compound with displacement ellipsoids drawn at the 50% probability level. H atoms are shown as spheres of arbitrary radius.

The mol­ecule of the title compound (Fig. 1 ▸) has a coumarin moiety at the C7 position and a penta­noate one at the O3 position. The dihedral angle between the coumarin nucleus and the penta­noate moiety is 62.20 (7)°. The coumarin moiety is planar with a maximum deviation from the least-squares plane of 0.081 (2) Å for atom O1. The bond distances and bond angles in the coumarin moiety are normal and are in good agreement with analogous structures (Rajalakshmi *et al.*, 1999 ▸; Anand Solomon *et al.*, 2003 ▸; Usman *et al.*, 2002 ▸; Krishna *et al.*, 2003 ▸; Kant *et al.*, 2004 ▸). The double-bond character of C1—O1 in the pyrone and C10—O4 in the penta­noate groups is confirmed by their distances of 1.2099 (15) and 1.2013 (16) Å, respectively. An inspection of the bond lengths shows that there is a slight asymmetry of the electronic distribution around the pyrone ring: the C1—C2 [1.4531 (17) Å] and C2—C3 [1.3441 (18) Å] bond lengths are respectively longer and shorter, than those excepted for a C_ar_—C_ar_ bond. This suggests that the electron density is preferentially located in the C2—C3 bond of the pyrone ring, as seen in other coumarin derivatives (Bationo *et al.*, 2024 ▸; Gomes *et al.*, 2016 ▸; Ouédraogo *et al.*, 2018 ▸). In addition, the bond angles, O2—C9—C8 and C3—C4—C5, at the junction of the pyrone and benzene rings are, respectively, smaller [116.5 (1)°] and greater [123.7 (1)°] than 120°. This phenomenon has also been observed in some analogous coumarins (Kanwal *et al.*, 2007 ▸).

**Figure 2:**
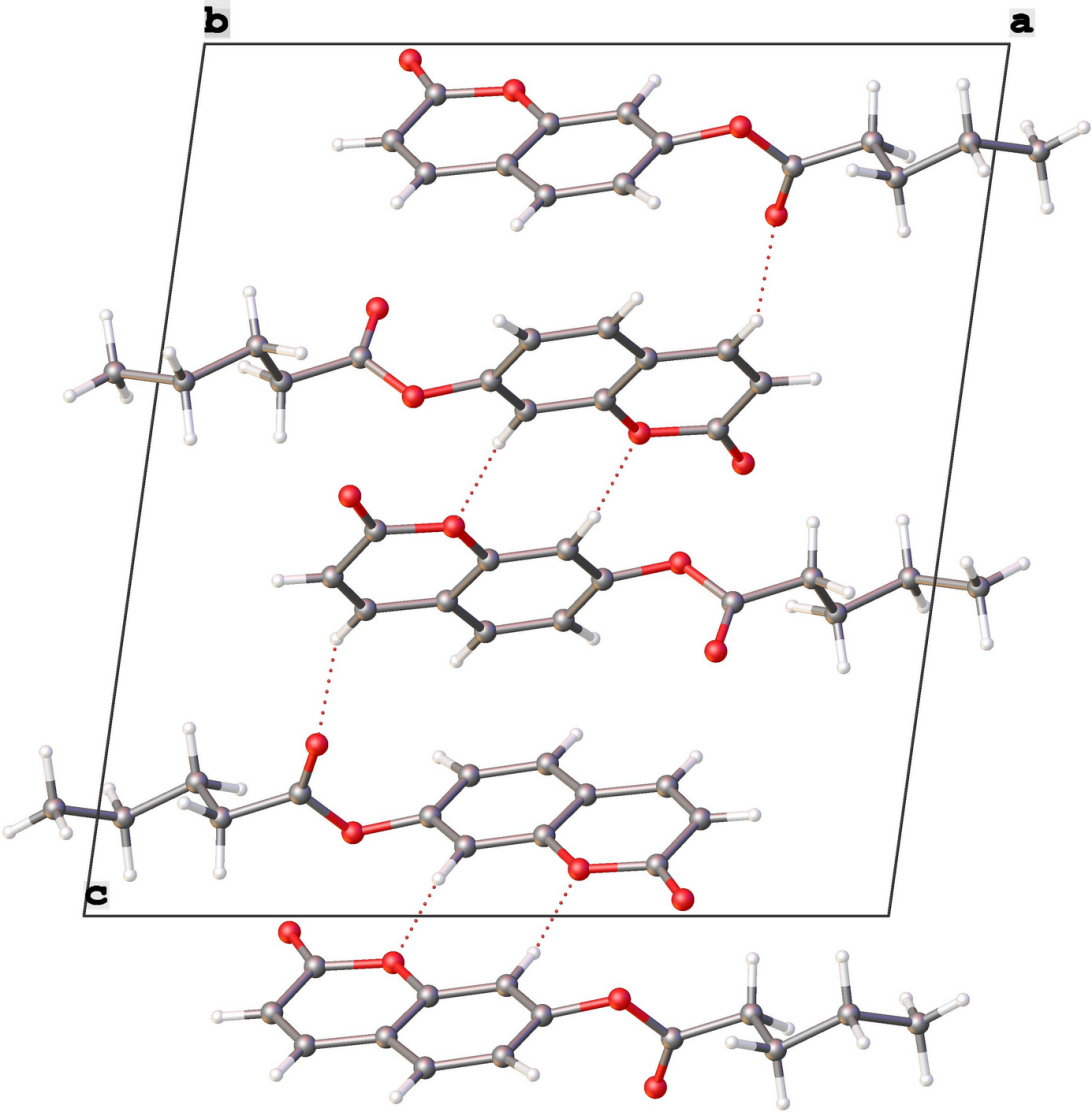
Part of the crystal structure viewed along the [010] direction. Hydrogen bonds are shown as dashed lines.

**Figure 3:**
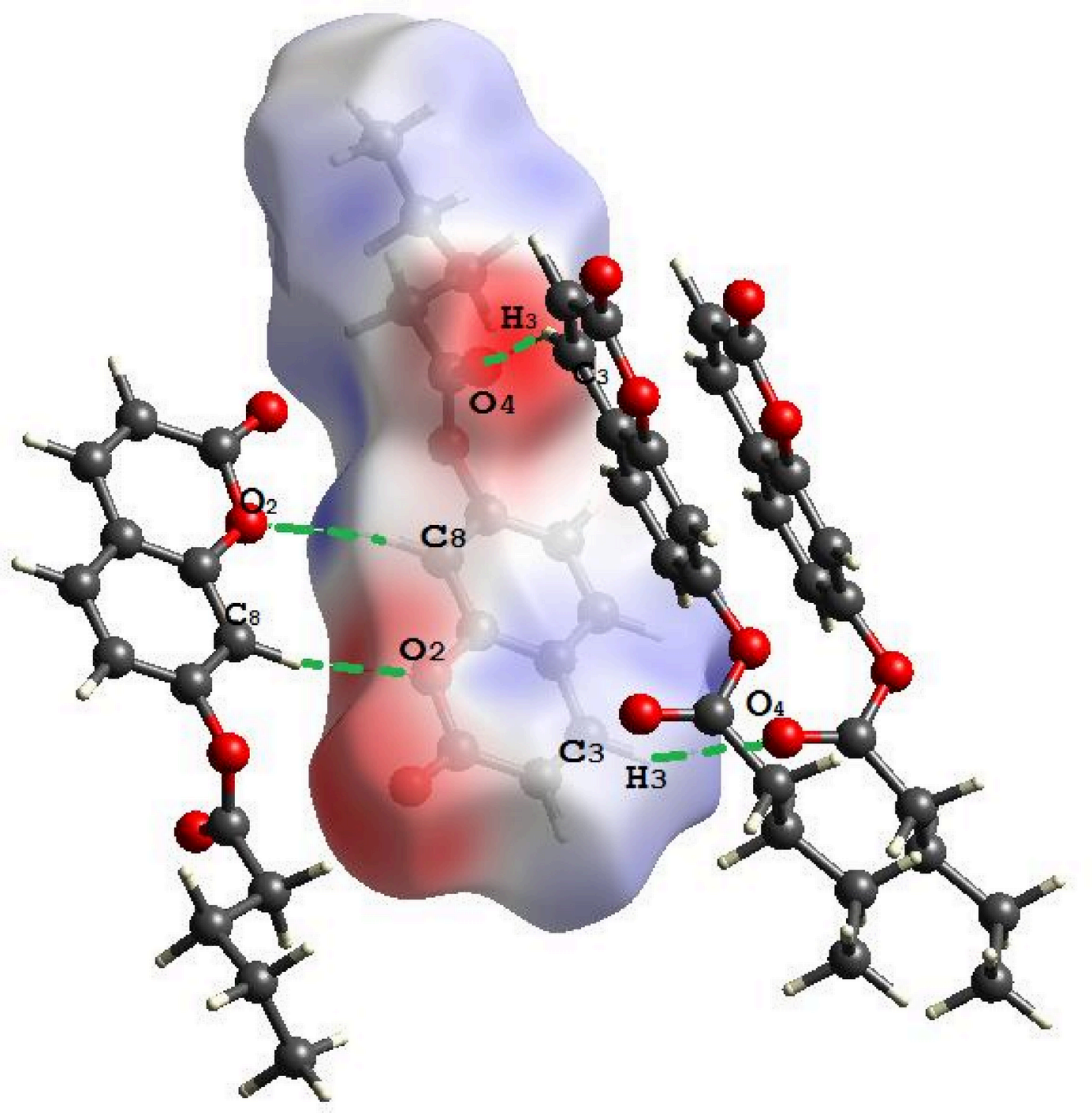
The Hirshfeld surface mapped over *d*_norm_ to visualize the inter­molecular contacts in the title compound.

**Figure 4:**
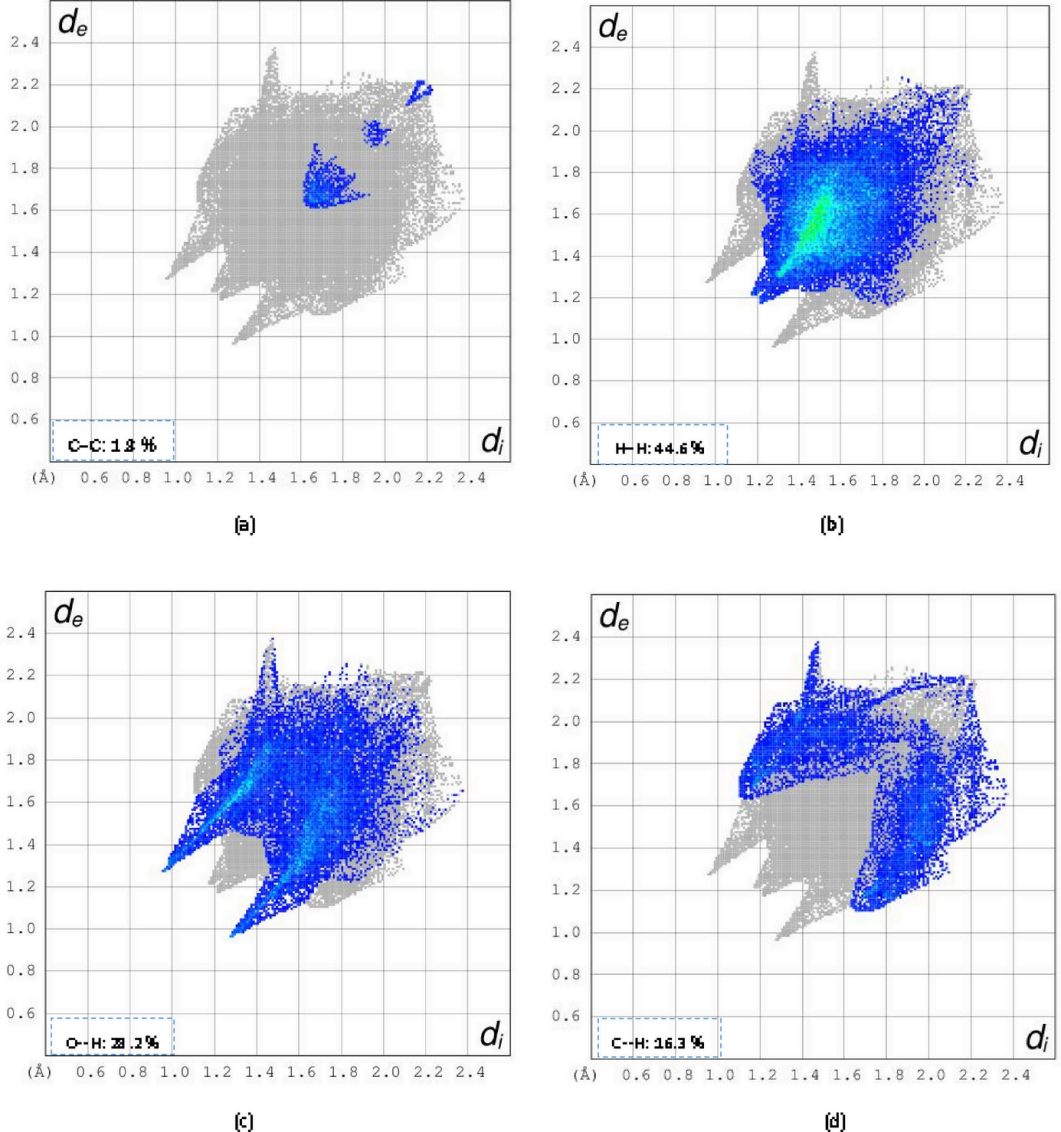
Fingerprint plots for the title compound showing (*a*) C⋯C, (*b*) H⋯H, (*c*) O⋯H/H⋯O and (*d*) C⋯H/H⋯C inter­actions. The outline of the full fingerprint is shown in grey. *d*_i_ is the closest inter­nal distance from a given point on the Hirshfeld surface and *d*_e_ is the closest external contact.

A view down along the [010] axis (Fig. 2 ▸) shows that in the crystal, mol­ecules are linked by C—O⋯H hydrogen bonds (Table 1 ▸) into centrosymmetric dimers with an 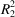
(8) graph-set motif (Etter *et al.*, 1990 ▸; Bernstein *et al.*, 1995 ▸). The cohesion of the crystal is also further supported by π–π inter­actions [*Cg*1⋯*Cg*1^i^ = 3.9342 (8) Å where *Cg*1 is the centroid of the C4–C9 ring; symmetry code: (i) 1 − *x*, 1 − *y*, 1 − *z*]. Another weak hydrogen bond inter­action is observed (C3—H3⋯O4; Table 2 ▸, Fig. 2 ▸). The inter­molecular inter­actions were qu­anti­fied using Hirshfeld surface analysis in order to visualize and understand them (Fig. 3 ▸). The two-dimensional fingerprint plots were generated with *CrystalExplorer 17* (Spackman *et al.*, 2021 ▸) to show the contribution of different inter­actions to the crystal cohesion (Fig. 4 ▸). Thus, 44.6% of the inter­molecular inter­actions are from H⋯H contacts, 28.2% are from H⋯O/O⋯H contacts and 16.3% are from H⋯C/C⋯H.

## Synthesis and crystallization

The title compound was synthesized by *O*-acyl­ation of umbelliferone with valeryl chloride (reagent) in the presence of diethyl ether as a solvent and pyridine as a base. To a solution of valeryl chloride (0.74 ml, 6.17 mmol, 1 equiv.) in dried diethyl ether (16 ml) were added dried pyridine (2.31 ml, 4.7 equiv.) and 7-hy­droxy­coumarin (1 g, 6.17 mmol, 1 equiv.) in small portions over 30 min, with vigorous stirring. The reaction mixture was left stirring at room temperature for 3 h. The resulting mixture was next poured in a separating funnel containing 40 ml of chloro­form and washed with diluted hydro­chloric acid solution until the pH was 2–3. The organic layer was extracted, washed with water to neutrality, dried with magnesium sulfate and the solvent removed *in vacuo*. The resulting crude product was washed with petroleum ether and recrystallized from chloro­form/*n*-hexane (1:3); the title compound, was thus obtained as a white powder (1.17 g, 77% yield). Colourless crystals suitable for single-crystal X-ray diffraction analysis were then formed from an acetone solution, after the solvent was left to evaporate slowly at room temperature. The melting point (338–340 K) was measured in open capillaries with a Cole-Parmer Stuart MP-800D Series- Melting Point S apparatus.

## Refinement

Crystal data, data collection and structure refinement details are summarized in Table 2 ▸.

## Supplementary Material

Crystal structure: contains datablock(s) I. DOI: 10.1107/S241431462500937X/bx4035sup1.cif

Structure factors: contains datablock(s) I. DOI: 10.1107/S241431462500937X/bx4035Isup3.hkl

Supporting information file. DOI: 10.1107/S241431462500937X/bx4035Isup3.cml

CCDC reference: 2497524

Additional supporting information:  crystallographic information; 3D view; checkCIF report

## Figures and Tables

**Table 1:** Hydrogen-bond geometry (Å, °)

*D*—H⋯*A*	*D*—H	H⋯*A*	*D*⋯*A*	*D*—H⋯*A*
C3—H3⋯O4^i^	0.95	2.37	3.243 (2)	153
C8—H8⋯O2^ii^	0.94	2.45	3.378 (2)	167

Symmetry codes: (i) 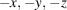; (ii) 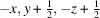.

**Table 2:** Experimental details

Crystal data	
Chemical formula	C_14_H_14_O_4_
*M*_r_	246.25
Crystal system, space group	Monoclinic, *P*2_1_/*c*
Temperature (K)	296
*a*, *b*, *c* (Å)	14.067 (2), 5.6449 (8), 15.400 (2)
β (°)	97.885 (5)
*V* (Å^3^)	1211.3 (3)
*Z*	4
Radiation type	Mo *K*α
μ (mm^−1^)	0.10
Crystal size (mm)	0.23 × 0.12 × 0.10

Data collection	
Diffractometer	Enraf–Nonius CAD4
No. of measured, independent and observed [*I* > 2σ(*I*)] reflections	75215, 4059, 3178
*R*_int_	0.052
(sin θ/λ)_max_ (Å^−1^)	0.737

Refinement	
*R*[*F*^2^ > 2σ(*F*^2^)], *wR*(*F*^2^), *S*	0.052, 0.154, 1.08
No. of reflections	4059
No. of parameters	219
H-atom treatment	All H-atom parameters refined
Δρ_max_, Δρ_min_ (e Å^−3^)	0.42, −0.32

Computer programs: *COLLECT* (Nonius, 1997 ▸), *DENZO*/*SCALEPACK* (Otwinowski & Minor, 1997 ▸), *SHELXT2018/2* (Sheldrick, 2015*a* ▸), *SHELXL2019/2* (Sheldrick, 2015*b* ▸), *CAMERON* (Watkin *et al.*, 1996 ▸) and *CRYSTALS* (Betteridge *et al.*, 2003 ▸).

## full crystallographic data

### 2-Oxo-2H-chromen-7-yl pentanoate Crystal data

**Table:**

C_14_H_14_O_4_	*F*(000) = 520
*M**_r_* = 246.25	*D*_x_ = 1.350 Mg m^−^^3^
Monoclinic, *P*2_1_/*c*	Mo *K*α radiation, λ = 0.71073 Å
*a* = 14.067 (2) Å	Cell parameters from 4060 reflections
*b* = 5.6449 (8) Å	θ = 2.9–31.6°
*c* = 15.400 (2) Å	µ = 0.10 mm^−^^1^
β = 97.885 (5)°	*T* = 296 K
*V* = 1211.3 (3) Å^3^	Prism, white
*Z* = 4	0.23 × 0.12 × 0.10 mm

### 2-Oxo-2H-chromen-7-yl pentanoate Data collection

**Table:**

Enraf–Nonius CAD4 diffractometer	*R*_int_ = 0.052
ω/2θ scans	θ_max_ = 31.6°, θ_min_ = 2.9°
75215 measured reflections	*h* = −20→19
4059 independent reflections	*k* = −8→8
3178 reflections with *I* > 2σ(*I*)	*l* = −22→22

### 2-Oxo-2H-chromen-7-yl pentanoate Refinement

**Table:**

Refinement on *F*^2^	Primary atom site location: structure-invariant direct methods
Least-squares matrix: full	Secondary atom site location: difference Fourier map
*R*[*F*^2^ > 2σ(*F*^2^)] = 0.052	Hydrogen site location: difference Fourier map
*wR*(*F*^2^) = 0.154	All H-atom parameters refined
*S* = 1.08	*w* = 1/[σ^2^(*F*_o_^2^) + (0.082*P*)^2^ + 0.3497*P*] where *P* = (*F*_o_^2^ + 2*F*_c_^2^)/3
4059 reflections	(Δ/σ)_max_ < 0.001
219 parameters	Δρ_max_ = 0.42 e Å^−^^3^
0 restraints	Δρ_min_ = −0.32 e Å^−^^3^

### 2-Oxo-2H-chromen-7-yl pentanoate Special details

**Table:**

Geometry. All esds (except the esd in the dihedral angle between two l.s. planes) are estimated using the full covariance matrix. The cell esds are taken into account individually in the estimation of esds in distances, angles and torsion angles; correlations between esds in cell parameters are only used when they are defined by crystal symmetry. An approximate (isotropic) treatment of cell esds is used for estimating esds involving l.s. planes.

### 2-Oxo-2H-chromen-7-yl pentanoate Fractional atomic coordinates and isotropic or equivalent isotropic displacement parameters (Å^2^)

**Table:**

	*x*	*y*	*z*	*U*_iso_*/*U*_eq_	
O2	0.39242 (6)	0.89521 (15)	0.55349 (5)	0.02545 (18)	
O3	0.67996 (6)	0.44508 (16)	0.59537 (6)	0.02665 (19)	
O1	0.25834 (7)	1.09807 (17)	0.51875 (7)	0.0345 (2)	
O4	0.73985 (7)	0.7127 (2)	0.69647 (7)	0.0460 (3)	
C9	0.44175 (8)	0.7035 (2)	0.59192 (7)	0.0229 (2)	
C8	0.53766 (8)	0.6840 (2)	0.57977 (7)	0.0242 (2)	
C2	0.25031 (8)	0.7636 (2)	0.61120 (8)	0.0282 (2)	
C6	0.54497 (9)	0.3126 (2)	0.66098 (7)	0.0261 (2)	
C7	0.58668 (8)	0.4856 (2)	0.61403 (7)	0.0239 (2)	
C1	0.29628 (8)	0.9302 (2)	0.55824 (8)	0.0268 (2)	
C4	0.39670 (8)	0.5355 (2)	0.63897 (7)	0.0235 (2)	
C5	0.45001 (9)	0.3398 (2)	0.67415 (7)	0.0259 (2)	
C3	0.29744 (8)	0.5745 (2)	0.64863 (8)	0.0269 (2)	
C11	0.84651 (9)	0.5030 (2)	0.61245 (9)	0.0301 (3)	
C10	0.75243 (9)	0.5724 (2)	0.64053 (8)	0.0295 (2)	
C12	0.88245 (10)	0.2667 (3)	0.65340 (11)	0.0386 (3)	
C13	0.96766 (11)	0.1684 (3)	0.61273 (12)	0.0428 (3)	
C14	1.05507 (11)	0.3292 (3)	0.62324 (13)	0.0453 (4)	
H6	0.5857 (12)	0.171 (3)	0.6822 (11)	0.035 (4)*	
H11A	0.8386 (13)	0.497 (3)	0.5494 (12)	0.039 (5)*	
H5	0.4193 (12)	0.219 (3)	0.7078 (10)	0.028 (4)*	
H12A	0.8271 (16)	0.147 (4)	0.6451 (14)	0.054 (6)*	
H2	0.1840 (14)	0.795 (3)	0.6158 (12)	0.043 (5)*	
H14A	1.1069 (16)	0.245 (4)	0.5961 (14)	0.058 (6)*	
H3	0.2666 (12)	0.460 (3)	0.6822 (10)	0.030 (4)*	
H11B	0.8947 (13)	0.636 (3)	0.6294 (12)	0.040 (5)*	
H12B	0.9014 (14)	0.299 (3)	0.7148 (13)	0.042 (5)*	
H8	0.5655 (13)	0.798 (3)	0.5427 (12)	0.041 (5)*	
H13A	0.9496 (15)	0.138 (4)	0.5470 (14)	0.056 (6)*	
H14B	1.0754 (16)	0.369 (4)	0.6879 (15)	0.061 (6)*	
H14C	1.0362 (16)	0.494 (4)	0.5950 (15)	0.065 (6)*	
H13B	0.9906 (15)	0.010 (4)	0.6454 (14)	0.059 (6)*	

### 2-Oxo-2H-chromen-7-yl pentanoate Atomic displacement parameters (Å^2^)

**Table:**

	*U*^11^	*U*^22^	*U*^33^	*U*^12^	*U*^13^	*U*^23^
O2	0.0241 (4)	0.0240 (4)	0.0288 (4)	0.0003 (3)	0.0057 (3)	0.0025 (3)
O3	0.0233 (4)	0.0290 (4)	0.0277 (4)	0.0005 (3)	0.0038 (3)	−0.0046 (3)
O1	0.0304 (5)	0.0319 (5)	0.0415 (5)	0.0054 (4)	0.0056 (4)	0.0066 (4)
O4	0.0295 (5)	0.0629 (7)	0.0458 (6)	−0.0041 (5)	0.0054 (4)	−0.0286 (5)
C9	0.0245 (5)	0.0233 (5)	0.0207 (4)	−0.0005 (4)	0.0025 (4)	−0.0015 (4)
C8	0.0250 (5)	0.0244 (5)	0.0235 (5)	−0.0020 (4)	0.0050 (4)	−0.0015 (4)
C2	0.0230 (5)	0.0333 (6)	0.0289 (5)	−0.0013 (4)	0.0056 (4)	0.0003 (4)
C6	0.0280 (5)	0.0270 (5)	0.0228 (5)	0.0003 (4)	0.0013 (4)	0.0007 (4)
C7	0.0231 (5)	0.0275 (5)	0.0211 (4)	−0.0004 (4)	0.0029 (4)	−0.0034 (4)
C1	0.0240 (5)	0.0279 (5)	0.0283 (5)	0.0013 (4)	0.0036 (4)	−0.0010 (4)
C4	0.0242 (5)	0.0256 (5)	0.0205 (4)	−0.0027 (4)	0.0029 (4)	−0.0002 (4)
C5	0.0277 (5)	0.0271 (5)	0.0225 (5)	−0.0023 (4)	0.0026 (4)	0.0020 (4)
C3	0.0256 (5)	0.0310 (6)	0.0245 (5)	−0.0045 (4)	0.0048 (4)	0.0002 (4)
C11	0.0243 (5)	0.0333 (6)	0.0329 (6)	0.0010 (4)	0.0053 (4)	−0.0003 (5)
C10	0.0248 (5)	0.0343 (6)	0.0290 (5)	0.0003 (4)	0.0028 (4)	−0.0033 (5)
C12	0.0275 (6)	0.0398 (7)	0.0491 (8)	0.0013 (5)	0.0072 (6)	0.0072 (6)
C13	0.0301 (6)	0.0366 (7)	0.0618 (10)	0.0018 (5)	0.0068 (6)	−0.0019 (7)
C14	0.0283 (7)	0.0473 (8)	0.0608 (10)	−0.0009 (6)	0.0083 (6)	−0.0030 (7)

### 2-Oxo-2H-chromen-7-yl pentanoate Geometric parameters (Å, º)

**Table:**

O2—C9	1.3750 (14)	C4—C3	1.4414 (16)
O2—C1	1.3787 (14)	C5—H5	0.991 (16)
O3—C10	1.3583 (15)	C3—H3	0.966 (17)
O3—C7	1.4002 (14)	C11—C10	1.4997 (17)
O1—C1	1.2099 (15)	C11—C12	1.531 (2)
O4—C10	1.2013 (16)	C11—H11A	0.962 (18)
C9—C8	1.3916 (16)	C11—H11B	1.020 (19)
C9—C4	1.3972 (15)	C12—C13	1.531 (2)
C8—C7	1.3810 (16)	C12—H12A	1.03 (2)
C8—H8	0.976 (19)	C12—H12B	0.965 (19)
C2—C3	1.3441 (18)	C13—C14	1.519 (2)
C2—C1	1.4531 (17)	C13—H13A	1.02 (2)
C2—H2	0.96 (2)	C13—H13B	1.06 (2)
C6—C5	1.3869 (17)	C14—H14A	1.01 (2)
C6—C7	1.3921 (16)	C14—H14B	1.02 (2)
C6—H6	1.011 (17)	C14—H14C	1.05 (2)
C4—C5	1.4014 (16)		
			
C9—O2—C1	122.05 (9)	C4—C3—H3	117.8 (10)
C10—O3—C7	117.85 (9)	C10—C11—C12	111.32 (11)
O2—C9—C8	116.47 (10)	C10—C11—H11A	108.3 (11)
O2—C9—C4	121.30 (10)	C12—C11—H11A	111.9 (11)
C8—C9—C4	122.22 (10)	C10—C11—H11B	108.4 (10)
C7—C8—C9	117.08 (10)	C12—C11—H11B	111.4 (10)
C7—C8—H8	122.5 (11)	H11A—C11—H11B	105.3 (15)
C9—C8—H8	120.0 (11)	O4—C10—O3	122.82 (12)
C3—C2—C1	121.55 (11)	O4—C10—C11	127.00 (12)
C3—C2—H2	122.8 (12)	O3—C10—C11	110.13 (11)
C1—C2—H2	115.6 (12)	C13—C12—C11	112.33 (12)
C5—C6—C7	118.76 (11)	C13—C12—H12A	109.5 (12)
C5—C6—H6	123.7 (10)	C11—C12—H12A	108.7 (12)
C7—C6—H6	117.5 (10)	C13—C12—H12B	110.1 (11)
C8—C7—C6	122.92 (11)	C11—C12—H12B	105.7 (11)
C8—C7—O3	119.14 (10)	H12A—C12—H12B	110.5 (17)
C6—C7—O3	117.74 (10)	C14—C13—C12	114.08 (14)
O1—C1—O2	116.98 (11)	C14—C13—H13A	107.0 (12)
O1—C1—C2	126.11 (11)	C12—C13—H13A	111.3 (12)
O2—C1—C2	116.90 (10)	C14—C13—H13B	105.4 (12)
C9—C4—C5	118.60 (10)	C12—C13—H13B	108.5 (12)
C9—C4—C3	117.68 (10)	H13A—C13—H13B	110.3 (17)
C5—C4—C3	123.72 (11)	C13—C14—H14A	107.2 (13)
C6—C5—C4	120.39 (11)	C13—C14—H14B	110.5 (13)
C6—C5—H5	119.8 (9)	H14A—C14—H14B	112.8 (17)
C4—C5—H5	119.8 (9)	C13—C14—H14C	109.3 (13)
C2—C3—C4	120.33 (11)	H14A—C14—H14C	113.8 (18)
C2—C3—H3	121.9 (10)	H14B—C14—H14C	103.3 (18)
			
C1—O2—C9—C8	177.80 (10)	C8—C9—C4—C5	−0.70 (16)
C1—O2—C9—C4	−0.89 (16)	O2—C9—C4—C3	−2.21 (16)
O2—C9—C8—C7	−176.65 (9)	C8—C9—C4—C3	179.17 (10)
C4—C9—C8—C7	2.03 (16)	C7—C6—C5—C4	1.46 (17)
C9—C8—C7—C6	−1.66 (17)	C9—C4—C5—C6	−1.10 (17)
C9—C8—C7—O3	173.10 (9)	C3—C4—C5—C6	179.04 (10)
C5—C6—C7—C8	−0.04 (17)	C1—C2—C3—C4	1.71 (18)
C5—C6—C7—O3	−174.88 (10)	C9—C4—C3—C2	1.74 (17)
C10—O3—C7—C8	78.01 (14)	C5—C4—C3—C2	−178.40 (11)
C10—O3—C7—C6	−106.95 (12)	C7—O3—C10—O4	1.68 (19)
C9—O2—C1—O1	−176.56 (10)	C7—O3—C10—C11	179.37 (10)
C9—O2—C1—C2	4.23 (16)	C12—C11—C10—O4	100.31 (17)
C3—C2—C1—O1	176.21 (12)	C12—C11—C10—O3	−77.26 (14)
C3—C2—C1—O2	−4.67 (17)	C10—C11—C12—C13	169.11 (12)
O2—C9—C4—C5	177.92 (10)	C11—C12—C13—C14	60.59 (19)

### 2-Oxo-2H-chromen-7-yl pentanoate Hydrogen-bond geometry (Å, º)

**Table:**

*D*—H···*A*	*D*—H	H···*A*	*D*···*A*	*D*—H···*A*
C3—H3···O4^i^	0.95	2.37	3.243 (2)	153
C8—H8···O2^ii^	0.94	2.45	3.378 (2)	167

Symmetry codes: (i) −*x*, −*y*, −*z*; (ii) −*x*, *y*+1/2, −*z*+1/2.
